# Synthesis and characterization of pyrrole-based group 4 PNP pincer complexes

**DOI:** 10.1007/s00706-024-03171-x

**Published:** 2024-02-09

**Authors:** Gerald Tomsu, Berthold Stöger, Karl Kirchner

**Affiliations:** 1 Institute of Applied Synthetic Chemistry, TU Wien, Getreidemarkt 9/163-AC, 1060 Vienna, Austria; 2 X-Ray Center, TU Wien, Getreidemarkt 9/163-AC, 1060 Vienna, Austria

**Keywords:** Pincer complexes, Pyrrole, Titanium, Zirconium, Hafnium

## Abstract

The synthesis, characterization, and reactivity of several group 4 metal complexes featuring a central anionic pyrrole moiety connected via CH_2_ linkers to two phosphine donors is described. Treatment of [P(NH)P-*i*Pr] with [MCl_4_(THF)_2_] (M = Zr, Hf) in the presence of base yields the dimeric complexes [M(PNP^*i*Pr^)(μ-Cl)(Cl)_2_]_2_ featuring two bridging chloride ligands. These complexes react with sodium cyclopentadienyl and SiMe_3_I to give the mononuclear complexes [M(PNP^*i*Pr^)(η^5^-Cp)(Cl)_2_] and [M(PNP^*i*Pr^)(I)_3_], respectively. The latter react with MeMgBr to form the trialkyl complexes [M(PNP^*i*Pr^)(Me)_3_]. Upon treatment of [Ti(NMe_2_)_4_] with [P(NH)P-*i*Pr] a complex with the general formula [Ti(PNP^*i*Pr^)(NMe_2_)_3_] is obtained. DFT calculations revealed that the most stable species is [Ti(κ^1^*N*- PNP^*i*Pr^)(NMe_2_)_3_] featuring a κ^1^*N*-bound PNP ligand. When [P(NH)P-*i*Pr] is reacted with [Ti(NMe_2_)_4_] in CH_2_Cl_2_ complex [Ti(PNP^*i*Pr^)(Cl)_2_(NMe_2_)] is formed. Treatment of a solution of [P(NH)P-*i*Pr] and [Zr(NMe_2_)_4_] with SiMe_3_Br affords the anionic seven-coordinate tetrabromo complex [Zr(PNP^*i*Pr^)(Br)_4_][H_2_NMe_2_]. The corresponding hafnium complex [Hf(PNP^*i*Pr^)(Br)_4_][H_2_NEt_2_] is obtained in similar fashion by utilizing [Hf(NEt_2_)_4_] as metal precursor. All complexes are characterized by means of NMR spectroscopy. Representative complexes were also characterized by X-ray crystallography.

**Graphical abstract:**

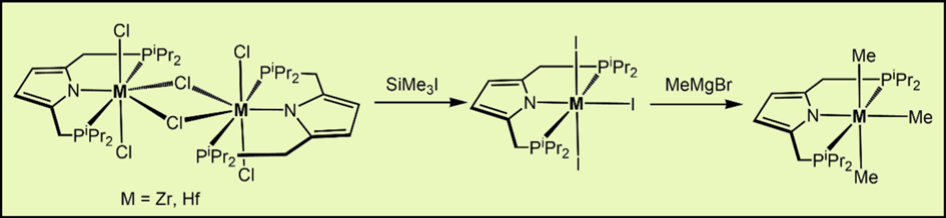

**Supplementary Information:**

The online version contains supplementary material available at 10.1007/s00706-024-03171-x.

## Introduction

Among the many types of transition metal complexes found in the chemical literature, pincer complexes play a particular role which have received tremendous attention for many decades [1–17]. The possibility of their rational and modular design enables, for instance, the generation of highly active catalysts for a range of chemical transformations with high selectivity. PCP pincer complexes, where the ligands bear phosphine donors tethered via CH_2_, O, or NR linkers to an aromatic anionic benzene backbone, are still one of the most common types. In the last couple of years pincer ligands which feature a monoanionic N-heterocyclic backbone, e.g., carbazole-, pyrrole-, and acridane-scaffolds, connected to phosphine (amido diphosphine PNP pincer ligands) have become an increasingly important class of compounds [18]. Within the large class of amido diphosphines, we are interested in PNP pincer ligands which feature a central anionic pyrrole moiety connected via CH_2_ linkers to two phosphine donors. These ligands are designed to form five-membered chelates upon coordination to a metal ion. The first transition metal complexes containing pyrrole-based PNP ligands were reported in 2012 independently by the groups of Gade [19], Mani [20], and Tonzetich [21]. Accordingly, a large number of transition metal complexes has been prepared to date using this class of ligand. With respect to group 4 metals pyrrole-based PNP pincer complexes are rare [22, 23]. We have recently described several Ti(IV) and Ti(III) PNP complexes [24] which were shown to undergo ketone insertion reactions into a Ti(IV)-P bond thereby forming new complexes with tridendate PNO-ligands.

Herein we report on the synthesis, characterization and reactivity of pyrrole-based M(IV) (M = Ti, Zr, Hf) PNP pincer complexes. Representative X-ray structures and DFT calculations are presented.

## Results and discussion

We have recently shown [24] that [P(NH)P-*i*Pr] (**1**) reacts with [TiCl_4_(THF)_2_] in the presence of base to yield the Ti(IV) complex [Ti(PNP^*i*Pr^)(Cl)_3_] (**2**). If the same reaction is performed with [MCl_4_(THF)_2_] (M = Zr, Hf), instead of monomeric analogs, the dimeric complexes [Zr(PNP^*i*Pr^)(μ-Cl)(Cl)_2_]_2_ (**2**) and [Hf(PNP^*i*Pr^)(μ-Cl)(Cl)_2_]_2_ (**3**) featuring two bridging chloride ligands are obtained in 76 and 80% isolated yields (Scheme 1). Noteworthy, the analogous zirconium complex featuring the bulkier PNP^*t*Bu^ ligand was reported by Nishibayashi and coworkers [22]. Complexes **2** and **3** were characterized ^1^H, ^13^C{^1^H}, and ^31^P{^1^H} NMR spectroscopy and elemental analysis. These complexes are highly symmetric as they display singlets at 40.2 and 42.8 ppm, respectively, in the ^31^P{^1^H} NMR spectrum. Likewise, in the ^1^H NMR spectrum the pyrrole hydrogen atoms give rise to singlets at 5.85 (2H) and 5.86 (2H) ppm. In the ^13^C{^1^H} NMR spectrum, the pyrrole carbons exhibit singlets at 138.3 and 137.9 ppm and 107.2 and 107.7 ppm assignable to the quaternary and tertiary carbon atoms, respectively.

**Figure:**
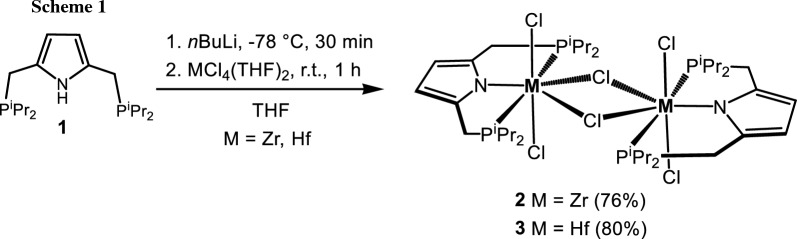

**Fig. 1:**
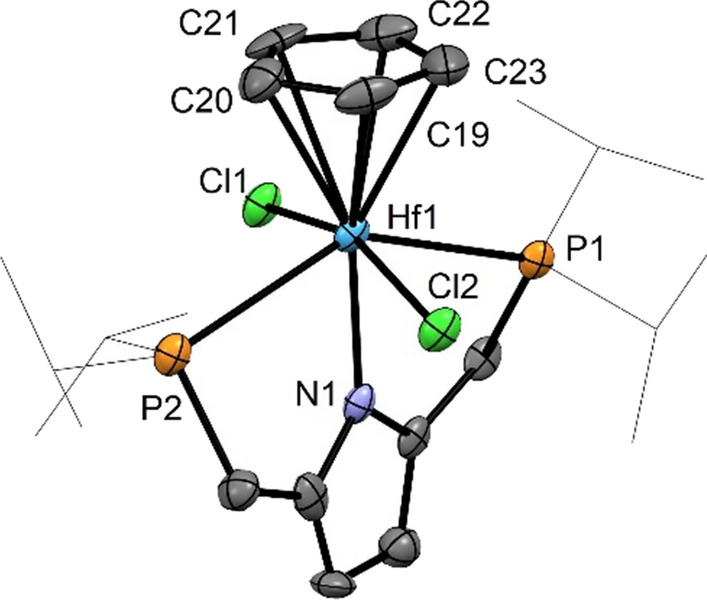
Structural view of [Hf(PNP^*i*Pr^)(η^5^-Cp)(Cl)_2_] (**5**) showing 50% thermal ellipsoids (H atoms are omitted for clarity). Selected bond lengths (Å) and bond angles (deg): Hf1-N1 2.237(7), Hf1-Cl2 2.484(2), Hf1-Cl1 2.485(3), Hf1-C22 2.51(1), Hf1-C23 2.54(1), Hf1-C21 2.54(1), Hf1-C19 2.54(1), Hf1-C20 2.55(1), Hf1-P1 2.751(2), Hf1-P2 2.771(3), Cl1-Hf1 Cl2 160.03(8), P1-Hf1-P2 139.39(7)

Reactions of [Zr(PNP^*i*Pr^)(μ-Cl)(Cl)_2_]_2_ (**2**) and [Hf(PNP^*i*Pr^)(μ-Cl)(Cl)_2_]_2_ (**3**) with 2 equiv of sodium cyclopentadienyl (CpNa) in THF at room temperature gives the corresponding mononuclear complexes [M(PNP^*i*Pr^)(η^5^-Cp)(Cl)_2_] (M = Zr (**4**), Hf (**5**)) in 90% and 93% yields, respectively (Scheme 2). These complexes were characterized by ^1^H, ^13^C{^1^H}, and ^31^P{^1^H} NMR spectroscopy. In the ^1^H NMR spectrum the Cp ligands of **4** and **5** exhibits triplet resonances at 6.70 (*J*_HP_ = 1.2 Hz) and 6.40 ppm (*J*_HP_ = 1.1 Hz), respectively. In the ^13^C{^1^H} NMR spectrum the Cp rings give rise to signals at 115.6 and 114.0 ppm. The molecular structure of **5** was confirmed by X-ray analysis. In addition, a structural view is shown in Fig. 1 with selected bond distances and angles reported in the caption. This complex adopts a five-legged piano-stool geometry around the hafnium center with the P, N, P atoms of the pyrrole moiety and the two chloride ligands as the legs. The analogous Zr complex with a PNP^*t*Bu^ ligand was reported recently [22].

**Figure:**
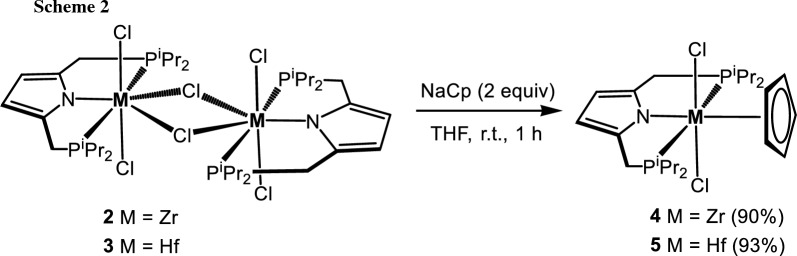

**Fig. 2:**
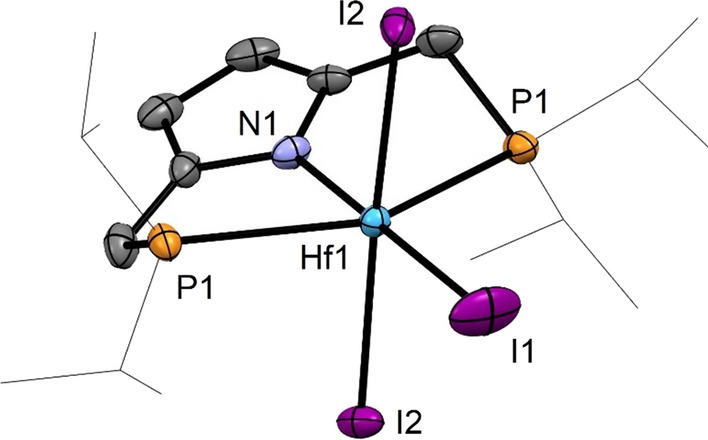
Structural view of [Hf(PNP^iPr^)(I)_3_] (**7**) showing 50% thermal ellipsoids (H atoms are omitted for clarity). Selected bond lengths (Å) and bond angles (deg): Hf1-N1 2.143(6), Hf1-P1 2.737(1), Hf1-I1 2.8051(8), Hf1-I2 2.7922(4), P1-Hf1-P1 145.21(6), I2-Hf1-I2 175.57(2), I2-Hf1-I1 87.78(1), N1-Hf1-I1 180.0°

Treatment of a solution of [Zr(PNP^*i*Pr^)(μ-Cl)(Cl)_2_]_2_ (**2**) and [Hf(PNP^*i*Pr^)(μ-Cl)(Cl)_2_]_2_ (**3**) in toluene at room temperature with an excess of SiMe_3_I afforded, after workup, the triiodide complexes [Zr(PNP^*i*Pr^)(I)_3_] (**6**) and [Hf(PNP^*i*Pr^)(I)_3_] (**7**) in 87 and 87%, respectively, isolated yields (Scheme 3). Crystals suitable for X-ray diffraction were obtained by layering a saturated CH_2_Cl_2_ solution of **7** with *n*-pentane. The solid-state structure of **7** was established by single-crystal X-ray diffraction. A molecular view is depicted in Fig. 2 with selected bond distances given in the captions. This complex has a distorted octahedral geometry with bond angles of 175.57(2)° (I1-Hf1-I2), 180.0° (N1-Hf1-I1), and 145.21(6)° (P1-Hf1-P1).

**Figure:**
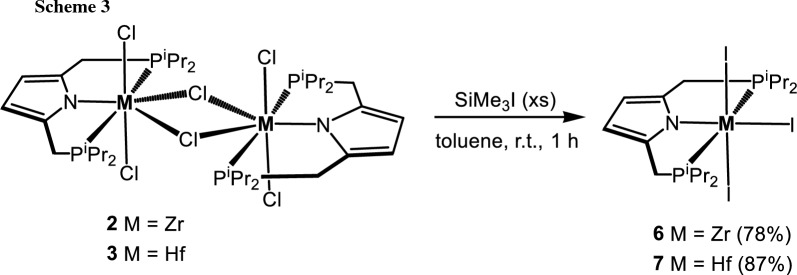

Complexes [Zr(PNP^*i*Pr^)(I)_3_] (**6**) and [Hf(PNP^*i*Pr^)(I)_3_] (**7**) are readily alkylated with MeMgBr affording [Zr(PNP^*i*Pr^)(Me)_3_] (**8**) and [Hf(PNP^*i*Pr^)(Me)_3_] (**9**) in 80 and 82% isolated yields (Scheme 4). These complexes again were characterized by ^1^H, ^13^C{^1^H}, and ^31^P{^1^H} NMR spectroscopy and elemental analysis. The Zr- and Hf-bound methyl groups of **8** and **9** give rise to one triplet resonance at 1.04 (*J*_HP_ = 3.8 Hz) and 0.74 ppm (*J*_HP_ = 3.9 Hz), respectively, in the ^1^H NMR spectrum. In the ^13^C{^1^H} spectrum, corresponding resonances at 60.6 and 59.4 ppm were observed. The equivalence of the three methyl groups is analogous to the observations for [M(PNP)(Me)_3_] (PNP = N(C_6_H_3_-*o*-Me-2-P*i*Pr_2_)_2_ and N(*o*-C_6_H_4_-2-P*i*Pr_2_)_2_, M = Zr, Hf) [25–27] and [Hf(PNP)(Me)_3_] (PNP = N(SiMe_2_CH_2_PR_2_)_2_, R = Me, *i*Pr, *t*Bu) [28] which is indicative of exchange among the methyl group sites that is rapid on the NMR timescale at ambient temperature.

**Figure:**
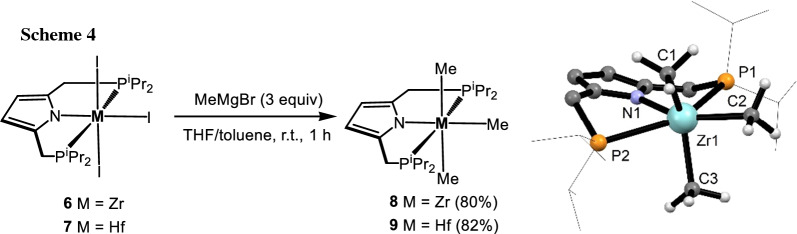

No static geometry can result in equivalent methyl groups in **8** and **9**. This can be seen from the DFT calculated structure of [Zr(PNP^*i*Pr^)(Me)_3_] (**8**) depicted in Scheme 4. The coordination environment about Zr in **8** can be described as a bicapped tetrahedron, with the two neutral P donors capping the faces of the N-Zr-Me_3_ tetrahedron. The ^1^H and ^13^C chemical shifts exhibited by the Me groups of **8** and **9** are comparable to those previously reported in similar compounds. For example, Ozerov’s and Liang’s [M(PNP)(Me)_3_] (M = Zr, Hf) and Fryzuk’s [Hf(PNP)(Me)_3_] compounds resonate in their ^1^H NMR spectra in the 0.5–0.9 ppm range [25–28].

**Fig. 3:**
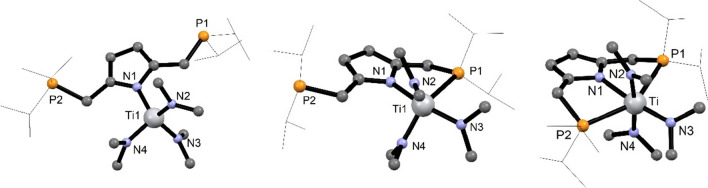
DFT calculated structures of **a** [Ti(κ^1^*N*-PNP^*i*Pr^)(NMe_2_)_3_] (**κ**^**1**^***N-10***), **b** [Ti(κ^2^*PN*-PNP^*i*Pr^)(NMe_2_)_3_] ((**κ**^**2**^***PN-10***), and **c** [Ti(κ^3^*PNP*-PNP^*i*Pr^)(NMe_2_)_3_] (**κ**^**3**^***PNP-10***). Free energies in kJ/mol for [M(PNP^*i*Pr^)(NMe_2_)_3_] (M = Ti, Zr, Hf) featuring κ^1^-, κ^2^-, and κ^3^-bound PNP ligands

Another strategy to afford Ti(IV) PNP complexes is the utilization of the amido-precursor [Ti(NMe_2_)_4_]. The targeted amido complex [Ti(PNP^*i*Pr^)(NMe_2_)_3_] (**10**) was considered to allow more functionalization possibilities in contrast to the above halide congeners. After stirring a solution of [P(NH)P-*i*Pr] (**1**) and 1 equiv of [Ti(NMe_2_)_4_] in toluene at 80 °C for 48 h, after workup, an amido complex tentatively assigned as [Ti(PNP^*i*Pr^)(NMe_2_)_3_] (**10**) was isolated as red oil in quantitative yield (Scheme 5). The ^1^H, ^13^C{^1^H}, and ^31^P{^1^H} NMR spectra of **10** revealed that this complex is highly symmetric in solution which is not in agreement with [Ti(PNP^*i*Pr^)(NMe_2_)_3_] (**10**) with the PNP ligand coordinated in κ^3^*PNP*-fashion. Singlet resonances were observed for the dimethylamido groups in the ^1^H and ^13^C{^1^H} NMR spectra at 3.14 and 44.6 ppm, respectively. Likewise, in the ^31^P{^1^H} NMR spectrum a singlet resonance is found at 6.2 ppm. A similar behavior was observed for the analogous Zr and Hf complexes and were thus not further investigated. The chemical equivalence of the NMe_2_ substituents and the phosphine moieties can be rationalized by isomerization reactions involving P-metal bond dissociation reactions. For titanium, DFT calculations revealed that the most stable species is [Ti(κ^1^*N*- PNP^*i*Pr^)(NMe_2_)_3_] (**κ**^**1**^***N-10***) featuring a κ^1^*N*-bound PNP ligand (Fig. 3). This compound is more stable by 36.6 and 87.5 kJ/mol, respectively, than the corresponding complexes with the PNP ligand being coordinated in κ^2^*PN*- and κ^3^*PNP*-fashion. This finding may suggest a fast equilibrium between **κ**^**1**^***N-10*** and **κ**^**2**^***PN-10*** in solution, whereas the formation of **κ**^**3**^***PNP-10*** seems to be unlikely. For comparison, in the case of Zr and Hf the energies of all three species are similar and may thus be in equilibrium with one another (Fig. 3). In fact, such a behavior was observed recently by Ballman and co-workers for [M(PNP)(NMe_2_)_3_] (PNP = N(CH_2_-*o*-C_6_H_4_PPh_2_)_2_ and N(C_6_H_4_-*o*-CH_2_PPh_2_)_2_), M = Zr, Hf). In addition, they were able to structurally characterize a κ^2^*PN*-bound hafnium complex [29].

**Figure:**
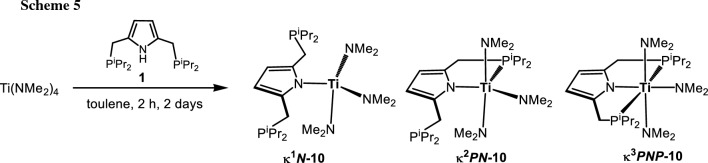

**Fig. 4:**
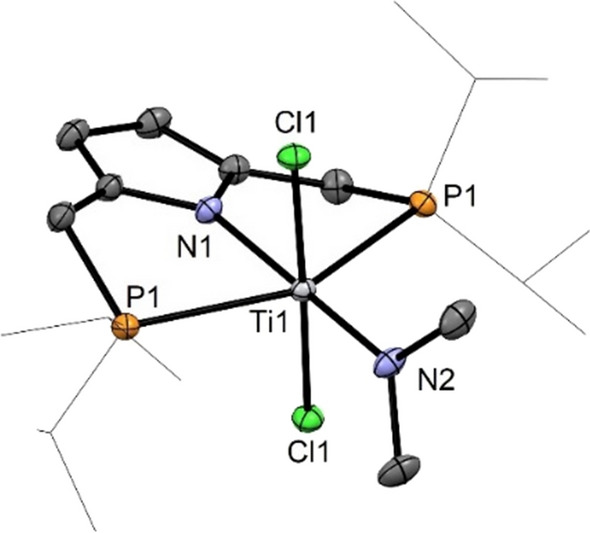
Structural view of [Ti(PNP^*i*Pr^)(Cl)_2_(NMe_2_)] (**11**) showing 50% thermal ellipsoids (H atoms are omitted for clarity). Selected bond lengths (Å) and bond angles (deg): Ti1-N1 2.114(1), Ti1-N2 1.9390(1), Ti1-Cl1 2.3419(3), Ti1-P1 2.5877(4), Cl1-Ti1-Cl1 177.34(2), P1-Ti1-P1 150.85(2), N1-Ti1-N2 180.0

Interestingly, when [P(NH)P-*i*Pr] (**1**) was reacted with 2 equivs of [Ti(NMe_2_)_4_] in CH_2_Cl_2_ at 40 °C complex [Ti(PNP^*i*Pr^)(Cl)_2_(NMe_2_)] (**11**) was obtained in 92% isolated yield (Scheme 6). This complex was fully characterized by ^1^H, ^13^C{^1^H}, and ^31^P{^1^H} NMR spectroscopy and elemental analysis. Additionally, the molecular structure of **11** was confirmed by X-ray analysis. A structural view is shown in Fig. 4 with selected bond distances and angles reported in the caption. The coordination geometry around the titanium center corresponds to a slightly distorted octahedron where the PNP ligand and the amide ligand define the equatorial plane and the two chloride ligands the axial positions. The two Ti-N bonds exhibit different bond distances being 2.114(1) Å for Ti1-N1 and 1.9390(1) Å Ti1-N2 which may be attributed to the fact that the dimethylamido ligand is both a stronger σ and π-donor than the nitrogen atom of the pyrrole moiety.

**Figure:**
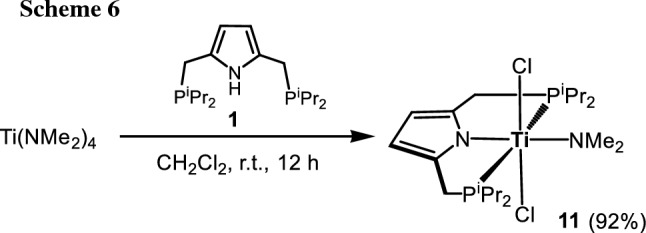

**Fig. 5:**
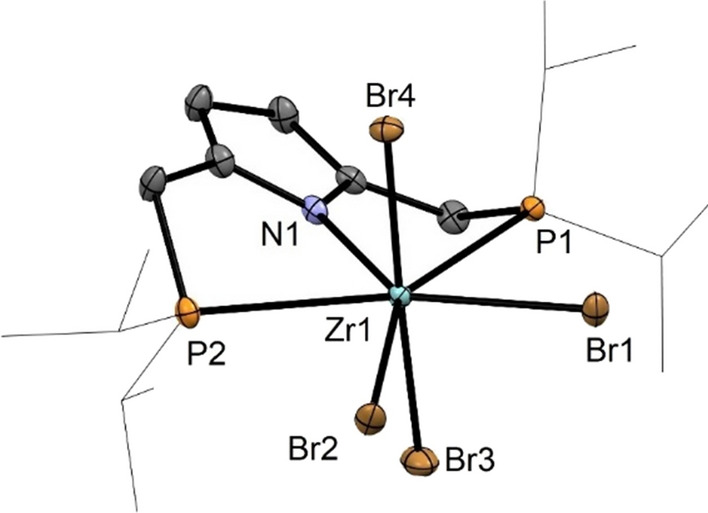
Structural view of [Zr(PNP^*i*Pr^)(Br)_4_][NH_2_Me_2_]⋅CH_2_Cl_2_ (**12**⋅CH_2_Cl_2_) showing 50% thermal ellipsoids (H atoms, the [NH_2_Me_2_].^+^ cation and CH_2_Cl_2_ are omitted for clarity). Selected bond lengths (Å) and bond angles (deg): Zr1-N1 2.251(4), Zr1-Br1 2.7763(7), Zr1-Br2 2.7352(7), Zr1-Br3 2.5865(7), Zr1-Br4 2.6323(7), Zr1-P1 2.772(1), Zr1-P2 2.785(1), Br3-Zr1-Br4 177.53(3), N1-Zr1-Br1 139.68(9), N1-Zr1-Br2 142.48(9), N1-Zr1-Br3 94.6(1) N1-Zr1-Br4 87.6(1) P1-Zr1-P2 135.33(4), Br2-Zr1-P1 148.96(3), Br1-Zr1-P2 150.87(3)

Treatment of a solution of [P(NH)P-*i*Pr] (**1**) and [Zr(NMe_2_)_4_] (1 equiv) in toluene at 120 °C for 72 h and subsequent addition of SiMe_3_Br (3.5 equiv) at room temperature afforded, after workup, the anionic seven coordinate tetrabromo complex [Zr(PNP^*i*Pr^)(Br)_4_][H_2_NMe_2_] (**13**) in 80% yield (Scheme 7). The corresponding hafnium complex [Hf(PNP^*i*Pr^)(Br)_4_][H_2_NEt_2_] (**14**) was obtained in similar fashion by utilizing [Hf(NEt_2_)_4_] as metal precursor. These complexes are very air and moisture sensitive. It has to be noted that, according to our knowledge, monomeric seven coordinate group 4 metal pincer complexes are unknown. In addition to the NMR spectroscopic characterization, the solid-state structure of **13** was determined by X-ray crystallography. A molecular view is depicted in Fig. 5 with selected bond distances given in the caption. The coordination sphere of the seven-coordinate Zr(IV) center may be described as a distorted pentagonal bipyramidal geometry.

**Figure:**
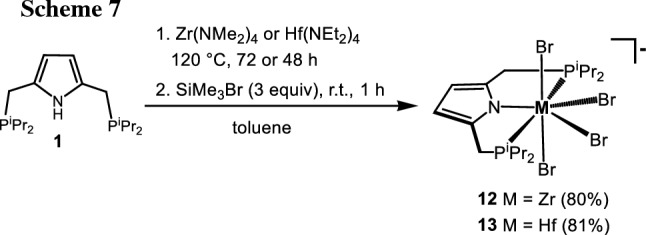

## Conclusion

In sum, we described the synthesis and reactivity of several new group 4 metal complexes containing a central anionic pyrrole moiety connected via CH_2_-linkers to two *i*Pr donors. As starting materials [MCl_4_(THF)_2_] (M = Zr, Hf) and [M(NMe_2_)_4_] (M = Ti, Zr) as well as [Hf(NEt_2_)_4_] were utilized. Treatment of [P(NH)P-*i*Pr] in the presence of base yields the dimeric complexes [M(PNP^*i*Pr^)(μ-Cl)(Cl)_2_]_2_ (M = Zr, Hf) featuring bridging chloride ligands. These dimeric complexes are precursors for several monomeric group 4 complexes including [M(PNP^*i*Pr^)(η^5^-Cp)(Cl)_2_] and [M(PNP^*i*Pr^)(I)_3_]. The latter react with MeMgBr to give trialkyl complexes of the type [M(PNP^iPr^)(Me)_3_]. Interestingly, if [P(NH)P-*i*Pr] is reacted with [M(NMe_2_)_4_] (M = Ti, Zr) and [Hf(NEt_2_)_4_] complexes of the type [M(PNP^*i*Pr^)(NMe_2_)_3_] and [Hf(PNP^*i*Pr^)(NEt_2_)_3_] were obtained. DFT calculations revealed that the most stable species is [Ti(κ^1^*N*-PNP^*i*Pr^)(NMe_2_)_3_] featuring a κ^1^*N*-bound PNP ligand. In solution, there is a fast equilibrium between complexes where the PNP ligand is coordinated in κ^1^*N*- and κ^2^*PN*-fashion. The formation of a species where the PNP ligand is coordinated in κ^3^*PNP-*fashion seems unlikely. On the other hand, in the case of Zr and Hf, the energies of all three species are similar and may thus be in equilibrium with one another. Finally, if a solution of [P(NH)P-*i*Pr] and [Zr(NMe_2_)_4_] was treated with SiMe_3_Br the anionic seven-coordinate tetrabromo complex [Zr(PNP^*i*Pr^)(Br)_4_][H_2_NMe_2_]. The corresponding hafnium complex [Hf(PNP^*i*Pr^)(Br)_4_][H_2_NEt_2_] was obtained in similar fashion by utilizing [Hf(NEt_2_)_4_] as metal precursor.

## Experimental

All manipulations were performed under an inert atmosphere of argon by using Schlenk techniques or in an MBraun inert-gas glovebox. The solvents were purified according to standard procedures [30]. The deuterated solvents were purchased from Eurisotop SAS and dried over 4 Å molecular sieves. The ligand precursor [P(NH)P-*i*Pr] (2,5-bis[[bis(1-methylethyl)phosphino]methyl]-1*H*-pyrrole, **1**) was prepared according to the literature [31]. All other starting materials are known compounds and were used as obtained from commercial sources. ^1^H, ^13^C{^1^H}, and ^31^P{^1^H} NMR spectra were recorded on Bruker AVANCE-250, AVANCE-400, and AVANCE-600 spectrometers. ^1^H and ^13^C{^1^H} NMR spectra were referenced internally to residual protio-solvent and solvent resonances, respectively, and are reported relative to tetramethylsilane (*δ* = 0 ppm). ^31^P{^1^H} NMR spectra were referenced externally to H_3_PO_4_ (85%) (*δ* = 0 ppm).

### Bis-[2,5-bis[[bis(1-methylethyl)phosphino-κ^2^*P*]methyl]-1*H*-pyrrolato-κ*N*](μ-chloro)(dichloro)zirconium(IV)], [Zr(PNP^*i*Pr^)(μ-Cl)(Cl)_2_]_2_ (2, C_36_H_68_Cl_6_N_2_P_4_Zr_2_)

A solution of [P(NH)P-*i*Pr] (**1**, 200 mg, 0.61 mmol) in THF (8 cm^3^) was treated with *n*-BuLi (419 mm^3^, 1.6 M in *n*-hexane, 0.67 mmol, 1.1 equiv.) at – 78 °C. After stirring for 30 min at this temperature the reaction mixture was allowed to reach room temperature and stirred for further 30 min and [ZrCl_4_(THF)_2_] (219 mg, 0.58 mmol, 0.95 equiv.) was added. Upon stirring for 1 h, all volatiles were removed under reduced pressure and the orange oily residue was redissolved in toluene (10 cm^3^). The orange solution was filtered through a syringe filter (PTFE, 0.2 µm), which was washed with toluene (2 × 8 cm^3^). After evaporation of the solvent, the residue was washed with *n*-pentane (3 × 10 cm^3^) until the washing phase was colorless. The product was obtained as beige powder. Yield: 230 mg (76%); ^1^H NMR (400 MHz, CD_2_Cl_2_, 25 °C): *δ* = 5.85 (s, 2H, Pyr^3,4^), 3.29–3.22 (m, 4H, CH_2_), 2.40–2.24 (m, 4H, C*H*CH_3_), 1.42–1.28 (m, 12H, CHC*H*_*3*_), 1.23–1.09 (m, 12H, CHC*H*_*3*_) ppm; ^13^C{^1^H} NMR (101 MHz, CD_2_Cl_2_, 25 °C): *δ* = 138.3 (C_q_, Pyr^2,5^), 107.2 (Pyr^3,4^), 25.0 (CH_2_), 19.2 (*C*HCH_3_), 18.3 (CH*C*H_3_) ppm; ^31^P{^1^H} NMR (162 MHz, CD_2_Cl_2_, 25 °C): *δ* = 40.2 ppm.

### Bis-[2,5-bis[[bis(1-methylethyl)phosphino-κ^2^*P*]methyl]-1*H*-pyrrolato-κ*N*](μ-chloro)(dichloro)hafnium(IV)], [Hf(PNP^*i*Pr^)(μ-Cl)(Cl)_2_]_2_ (3, C_36_H_68_Cl_6_Hf_2_N_2_P_4_)

This complex was prepared analogously to **2** with [P(NH)P-*i*Pr] (**1**, 200 mg, 0.61 mmol), *n*-BuLi (419 mm^3^, 1.6 M in hexane, 0.67 mmol, 1.1 equiv.) and [HfCl_4_(THF)_2_] (283 mg, 0.58 mmol, 0.95 equiv.) as starting materials. Yield: 298 mg (80%). Single crystals for X-ray diffraction measurement were obtained by layering a saturated CH_2_Cl_2_ solution with *n*-pentane. ^1^H NMR (600 MHz, CD_2_Cl_2_, 25 °C): *δ* = 5.86 (s, 2H), 3.28 (s, 4H), 2.34 (bs, 4H), 1.39–1.31 (m, 12H), 1.18 (bs, 12H) ppm; ^13^C{^1^H} NMR (151 MHz, CD_2_Cl_2_, 25 °C): *δ* = 137.9 (C_q_, Pyr^2,5^), 107.7 (Pyr^3,4^), 24.9 (*C*HCH_3_), 21.0 (CH_2_), 19.3 (CH*C*H_3_), 18.4 (CH*C*H_3_) ppm; ^31^P{^1^H} NMR (243 MHz, CD_2_Cl_2_, 25 °C): *δ* = 42.8 ppm.

###  [2,5-Bis[[bis(1-methylethyl)phosphino-κ^2^*P*]methyl]-1*H*-pyrrolato-κ*N*](η^5^-cyclopentadienyl)(dichloro)zirconium(IV)], [Zr(PNP^*i*Pr^)(η^5^-Cp)(Cl)_2_] (4, C_23_H_39_Cl_2_NP_2_Zr)

To a solution of **2** (100 mg, 0.095 mmol) in THF (6 cm^3^) NaCp (79.5 mm^3^, 2.4 M in THF, 0.19 mmol, 2 equiv.) was added at room temperature whereupon the solution became immediately dark red. Upon stirring for 1 h, the solvent was evaporated and the residue was redissolved in toluene (10 cm^3^). The solution was filtered through a syringe filter (PTFE, 0.2 µm). Upon evaporation of the solvent and washing of the residue with *n*-pentane (2 × 10 cm^3^) the product was obtained as brown powder. Yield: 95 mg (90%); ^1^H NMR (600 MHz, CD_2_Cl_2_, 25 °C): *δ* = 6.70 (t, *J* = 1.2 Hz, 5H, Cp), 5.78 (s, 2H, Pyr^3,4^), 3.20–3.14 (m, 4H, CH_2_), 2.34–2.26 (m, 4H, C*H*CH_3_), 1.32–1.28 (m, 12H, CHC*H*_*3*_), 1.28–1.23 (m, 12H, CHC*H*_*3*_) ppm; ^13^C{^1^H} NMR (151 MHz, CD_2_Cl_2_ 25 °C): *δ* = 135.0 (t, *J* = 4.3 Hz, C_q_, Pyr^2,5^), 115.6 (Cp), 105.8 (t, *J* = 4.5 Hz, Pyr^3,4^), 25.5 (t, *J* = 5.9 Hz, C*H*CH_3_), 25.3 (dd, *J* = 9.5, 8.0 Hz, CH_2_), 19.6 (d, *J* = 7.9 Hz, CH*C*H_3_) ppm; ^31^P{^1^H} NMR (243 MHz, CD_2_Cl_2_, 25 °C): *δ* = 36.2 ppm.

### [2,5-Bis[[bis(1-methylethyl)phosphino-κ^2^*P*]methyl]-1*H*-pyrrolato-κ*N*](η^5^-cyclopentadienyl)(dichloro)hafnium(IV)], [Hf(PNP^*i*Pr^)(η^5^-Cp)(Cl)_2_] (5, C_23_H_39_Cl_2_HfNP_2_)

This complex was prepared analogously to **4** with **3** (250 mg, 0.20 mmol) and NaCp (170 mm^3^, 2.4 M in THF, 0.40 mmol, 2 equiv) as starting materials. Yield: 243 mg (93%). Single crystals for X-ray diffraction measurements were obtained by layering a saturated CH_2_Cl_2_ solution with *n*-pentane. ^1^H NMR (600 MHz, CD_2_Cl_2_, 25 °C): *δ* = 6.40 (t, *J* = 1.1 Hz, 5H, Cp), 5.64 (s, 2H, Pyr^3,4^), 3.11–3.00 (m, 4H, CH_2_), 2.25–2.16 (m, 4H, C*H*CH_2_), 1.19–1.15 (m, 12H, CHC*H*_*3*_), 1.15–1.11 (m, 12H, CHC*H*_*3*_) ppm; ^13^C{^1^H} NMR (151 MHz, CD_2_Cl_2_, 25 °C): *δ* = 134.9 (t, *J* = 4.2 Hz, C_q_, Pyr^2,5^), 114.0 (Cp), 106.2 (t, *J* = 4.4 Hz, Pyr^3,4^), 25.5 (t, *J* = 6.8 Hz, *C*HCH_3_), 25.1 (d, *J* = 8.9 Hz, CH_2_), 25.0 (d, *J* = 9.0 Hz, CH_2_), 24.2 (d, 13.5 Hz, CH*C*H_3_), 19.6 (CH*C*H_3_) ppm; ^31^P{^1^H} NMR (243 MHz, CD_2_Cl_2_, 25 °C): *δ* = 37.8 ppm.

### [2,5-Bis[[bis(1-methylethyl)phosphino-κ^2^*P*]methyl]-1*H*-pyrrolato-κ*N*](triiodo)zirconium(IV)], [Zr(PNP^*i*Pr^)(I)_3_] (6, C_18_H_34_I_3_NP_2_Zr)

To a solution of **2** (100 mg, 0.095 mmol) in toluene (10 cm^3^) SiMe_3_I (407 mm^3^, 2.86 mmol, 30 equiv.) was added at room temperature and stirred for 1 h. During addition of SiMe_3_I the solution became orange and a precipitate was formed. The solution was decanted and the remaining residue was extracted three times with toluene (10 cm^3^). The organic layers were combined and all volatiles were removed under reduced pressure. The orange residue was washed with *n*-pentane (3 × 10 cm^3^) affording **6** as orange powder. Yield: 120 mg (78%); ^1^H NMR (400 MHz, CD_2_Cl_2_, 25 °C): *δ* = 5.88 (s, 2H, Pyr^3,4^), 3.48–3.32 (m, 4H, CH_2_), 2.63–2.48 (m, 4H, C*H*CH_3_), 1.45–1.18 (m, 24H, CHC*H*_*3*_) ppm; ^13^C{^1^H} NMR (101 MHz, CD_2_Cl_2_, 25 °C): *δ* = 139.1 (t, *J* = 5.3 Hz, Pyr^2,5^), 108.6 (t, *J* = 4.3 Hz, Pyr^3,4^), 27.2 (t, *J* = 7.0 Hz, *C*HCH_3_), 26.7 (t, *J* = 9.9 Hz, CH_2_), 20.1 (CH*C*H_3_), 19.7 (CH*C*H_3_) ppm; ^31^P{^1^H} NMR (162 MHz, CD_2_Cl_2_, 25 °C): *δ* = 56.5 ppm.

### [2,5-Bis[[bis(1-methylethyl)phosphino-κ^2^*P*]methyl]-1*H*-pyrrolato-κ*N*](triiodo)hafnium(IV)], [Hf(PNP^*i*Pr^)(I)_3_] (7, C_18_H_34_HfI_3_NP_2_)

This complex was prepared analogously to **6** with **3** (100 mg, 0.082 mmol) and SiMe_3_I (249 mm^3^, 2.5 mmol, 30 equiv.) as starting materials. Yield: 57 mg (87%). Single crystals for X-ray measurements were obtained by layering a saturated CH_2_Cl_2_ solution with *n-*pentane. ^1^H NMR (400 MHz, CH_2_Cl_2_, 25 °C): *δ* = 5.84 (s, 2H, Pyr^3,4^), 3.56–3.40 (m, 4H, CH_2_), 2.73–2.49 (m, 4H, CHC*H*_*3*_), 1.38 (ddt, *J* = 10.2, 7.1, 3.6 Hz, 24H, CHC*H*_*3*_) ppm; ^13^C{^1^H} NMR (101 MHz, CD_2_Cl_2_, 25 °C): *δ* = 139.0 (t, *J* = 4.8 Hz, Pyr^2,5^, C_q_, Pyr^2,5^), 109.4 (t, *J* = 4.3 Hz, Pyr^3,4^), 27.5 (t, *J* = 9.9 Hz, CH_2_), 27.2 (t, *J* = 8.2 Hz, *C*HCH_3_), 20.1 (d, *J* = 6.4 Hz, CH*C*H_3_) ppm; ^31^P{^1^H} NMR (162 MHz, CD_2_Cl_2_, 25 °C): *δ* = 62.3 ppm.

### [2,5-Bis[[bis(1-methylethyl)phosphino-κ^2^*P*]methyl]-1*H*-pyrrolato-κ*N*](trimethyl)zirconium(IV)], [Zr(PNP^*i*Pr^)(Me)_3_] (8, C_21_H_43_NP_2_Zr)

A suspension of **6** (60 mg, 0.075 mmol) in toluene (5 cm^3^) was treated with MeMgBr (0.23 mmol, 161 mm^3^, 1.4 M, 3 equiv) in THF/toluene (1:4) at room temperature. During the addition of MeMgBr a clear solution was formed. Dioxane (116 mm^3^, 1.35 mmol, 6 equiv) was added for precipitation of magnesia salts. Upon stirring for 1 h, all volatiles were evaporated under reduced pressure and the white residue was redissolved in *n*-pentane (5 cm^3^). The reaction mixture was filtered through a syringe filter (PTFE, 0.2 mm^3^) to afford a pale orange solution. After evaporation of the solvent the product was obtained as orange oil. Yield: 28 mg (80%); ^1^H NMR (400 MHz, C_6_D_6_, 25 °C): *δ* = 6.26 (d, *J* = 0.9 Hz, 2H, Pyr^3,4^), 2.90 (d, *J* = 6.1 Hz, 4H, CH_2_), 1.94 (dq, *J* = 14.4, 7.2 Hz, 2H, C*H*CH_3_), 1.04 (t, *J* = 3.8 Hz, 9H, Zr-CH_3_), 0.99 (dd, *J* = 13.6, 7.1 Hz, 12H, CHC*H*_*3*_), 0.92 (dd, *J* = 12.8, 7.1 Hz, 12H, CHC*H*_*3*_) ppm; ^13^C{^1^H} NMR (101 MHz, C_6_D_6_): *δ* = 136.6 (t, *J* = 6.3 Hz, C_q_, Pyr^4,5^), 107.0 (t, *J* = 4.3 Hz, Pyr^3,4^), 51.7 (Zr-CH_3_), 23.9–23.4 (m, CH_2_), 23.3–23.0 (m, *C*HCH_3_), 18.3 (d, *J* = 7.4 Hz, CH*C*H_3_) ppm; ^31^P{^1^H} NMR (162 MHz, C_6_D_6_, 25 °C): *δ* = 27.9 ppm.

### [2,5-Bis[[bis(1-methylethyl)phosphino-κ^2^*P*]methyl]-1*H*-pyrrolato-κ*N*](trimethyl)hafnium(IV)], [Hf(PNP^*i*Pr^)(Me)_3_] (9, C_21_H_43_HfNP_2_)

This complex was prepared analogously to **8** with **7** (60 mg, 0.067 mmol) and MeMgBr (0.20 mmol, 145 mm^3^, 1.4 M, 3.5 equiv) as starting materials. Yield: 31 mg (82%); ^1^H NMR (400 MHz, C_6_D_6_, 25 °C): *δ* = 6.24 (s, 2H, Pyr^3,4^), 2.93 (d, *J* = 5.6 Hz, 4H, CH_2_), 2.05–1.88 (m, *J* = 7.2 Hz, 4H, C*H*CH_3_), 1.02–0.86 (m, 24H, CHC*H*_*3*_), 0.74 (t, *J* = 3.9 Hz, 9H, Hf-CH_3_) ppm; ^13^C{^1^H} NMR (101 MHz, C_6_D_6_, 25 °vC): *δ* = 136.5–136.1 (m, Pyr^2,5^), 106.8–106.6 (m, Pyr^3,4^), 59.4 (t, *J* = 6.4 Hz, Hf-CH_3_), 22.6 (d, *J* = 7.1 Hz, CH_2_), 22.3 (d, *J* = 10.1 Hz, C*H*CH_3_), 17.6 (dt, *J* = 18.1, 1.2 Hz, CH*C*H_3_) ppm; ^31^P{^1^H} NMR (162 MHz, C_6_D_6_, 25 °C): *δ* = 30.8 ppm.

### Reaction of tetrakis(dimethylamido)titanium(IV), ([Ti(NMe_2_)_4_]), with (2,5-bis[[bis(1-methylethyl)phosphino]methyl]-1*H*-pyrrole), [P(NH)P-*i*Pr] (1). Formation of [2,5-bis[[bis(1-methylethyl)phosphino]methyl]-1*H*-pyrrolato]tris-(dimethylamido)titanium(IV)], [Ti(PNP^iPr^)(NMe_2_)_3_] (10)

A solution of [P(NH)P-*i*Pr] (**1**, 100 mg, 0.31 mmol) and [Ti(NMe_2_)_4_] (71 mm^3^, 0.31 mmol) in toluene (4 cm^3^) was stirred for 2 days at 80 °C. After removing of all volatiles under reduced pressure, **10** was obtained as red oil. ^1^H NMR (400 MHz, C_6_D_6_, 25 °C): *δ* = 6.42 (s, 2H, Pyr^3,4^), 3.14 (s, 18H, NCH_3_), 2.82–2.77 (m, 4H, CH_2_), 1.90–1.64 (m, 4H, C*H*CH_3_), 1.08 (dd, *J* = 7.1, 3.6 Hz, 12H, CHC*H*_*3*_), 1.05 (dd, *J* = 7.1, 2.3 Hz, 12H, CHC*H*_*3*_) ppm; ^13^C{^1^H} NMR (101 MHz, C_6_D_6_, 25 °C): *δ* = 136.7 (d, *J* = 12.4 Hz, C_q_, Pyr^2,5^), 107.7 (d, *J* = 4.9 Hz, Pyr^3,4^), 44.6 (CH_3_), 24.8 (d, *J* = 11.7 Hz, CH_2_), 24.3 (d, *J* = 14.8 Hz, *C*HCH_3_), 20.4 (d, *J* = 14.8 Hz, CH*C*H_3_), 19.5 (d, *J* = 10.6 Hz, CH*C*H_3_) ppm; ^31^P{^1^H} NMR (162 MHz, C_6_D_6_, 25 °C): *δ* = 6.2 ppm.

### [2,5-Bis[[bis(1-methylethyl)phosphino-κ^2^*P*]methyl]-1*H*-pyrrolato-κ*N*](dichloro)(dimethylamido)titanium(IV)], [Ti(PNP^*i*Pr^)(Cl)_2_(NMe_2_)] (11, C_20_H_40_Cl_2_N_2_P_2_Ti)

A solution of [P(NH)P-*i*Pr] (**1**, 100 mg, 0.31 mmol) and [Ti(NMe_2_)_4_] (142 mm^3^, 0.62 mmol, 2 equiv.) in CH_2_Cl_2_ (5 cm^3^) was stirred for 12 h at room temperature. After removing of all volatiles under reduced pressure, the product was obtained as brown solid. Yield: 140 mg (92%). Single crystals for X-ray diffraction measurements could be obtained from a saturated *n*-pentane solution at – 20 °C. ^1^H NMR (400 MHz, CD_2_Cl_2_, 25 °C): *δ* = 6.40 (Pyr^3,4^), 3.13–3.04 (m, 4H, CH_2_), 2.50 (s, 6H, NC*H*_*3*_), 1.88–1.60 (m, 4H, C*H*CH_3_), 1.45–1.15 (m, 24H, CHC*H*_*3*_) ppm; ^13^C{^1^H} NMR (101 MHz, CD_2_Cl_2_, 25 °C): *δ* = 135.2 (Pyr^2,5^), 105.9 (Pyr^3,4^), 44.5 (CH_3_), 24.8 (CH_2_), 20.2 (CH*C*H_3_), 18.4 (CH*C*H_3_) ppm; ^31^P{^1^H} NMR (162 MHz, C_6_D_6_, 25 °C): *δ* = 54.0 ppm.

### Dimethylammonium [2,5-bis[[bis(1-methylethyl)phosphino-κ^2^*P*]methyl]-1*H*-pyrrolato-κ*N*](tetrabromo)zirconium(IV)], [Zr(PNP^*i*Pr^)(Br)_4_][NH_2_Me_2_] (12, C_20_H_42_Br_4_N_2_P_2_Zr)

A solution of [P(NH)P-*i*Pr] (**1**, 200 mg, 0.61 mmol) and [Zr(NMe_2_)_4_] (163 mg, 0.61 mmol) in toluene (5 cm^3^) was stirred at 120 °C for 72 h. The reaction mixture was then allowed to reach room temperature and SiMe_3_Br (282 mm^3^, 2.1 mmol, 3.5 equiv) was added. After 5 min an orange precipitate was formed. All volatiles were removed under reduced pressure and the residue was washed with *n*-pentane (4 × 10 cm^3^). The product was obtained as orange powder. Yield: 383 mg (80%). Single crystals for X-ray diffraction measurement were obtained by layering a saturated CH_2_Cl_2_ solution with *n*-pentane. ^1^H NMR (400 MHz, CD_2_Cl_2_, 25 °C): *δ* = 7.64 (bs, 2H, *H*_2_NMe_2_), 5.90 (s, 2H, Pyr^3,4^), 3.27 (d, *J* = 6.4 Hz, 4H, CH_2_), 2.94 (s, 6H, Zr-N-CH_3_), 2.58–2.40 (m, 4H, C*H*CH_3_), 1.34 (dd, *J* = 13.5, 7.2 Hz, 12H, CHC*H*_*3*_), 1.24 (dd, *J* = 12.0, 7.1 Hz, 12H, CHC*H*_*3*_) ppm; ^13^C{^1^H} NMR (101 MHz, CD_2_Cl_2_, 25 °C): *δ* = 137.6 (t, *J* = 5.6 Hz, C_q_, Pyr^2,5^), 106.3 (Pyr^3,4^), 36.2 (Zr-N-CH_3_), 24.9 (t, *J* = 5.2 Hz), 21.4 (*C*HCH_3_), 18.9 (CH*C*H_3_), 18.0 (CH*C*H_3_) ppm; ^31^P{^1^H} NMR (162 MHz, CD_2_Cl_2_, 25 °C): *δ* = 39.7 ppm.

### Diethylammonium [2,5-bis[[bis(1-methylethyl)phosphino-κ^2^*P*]methyl]-1*H*-pyrrolato-κ*N*](tetrabromo)hafnium(IV)], [Hf(PNP^*i*Pr^)(Br)_4_][NH_2_Et_2_] (13, C_22_H_46_Br_4_HfN_2_P_2_)

A solution of [P(NH)P-*i*Pr] (**1**, 200 mg, 0.61 mmol) and [Hf(NEt_2_)_4_] (228 mm^3^, 0.61 mmol) in toluene (5 cm^3^) was stirred at 120 °C for 48 h. The reaction mixture was allowed to reach room temperature and SiMe_3_Br (282 mm^3^, 2.12 mmol, 3.5 equiv.) was added. After stirring for 1 h the precipitate was filtered through a syringe filter (PTFE, 0.2 mm^3^) and washed with toluene (3 × 10 cm^3^). All volatiles were removed under reduced pressure. After washing of the residue with *n*-pentane (10 cm^3^) the product was obtained as orange powder. Yield: 390 mg (81%); ^1^H NMR (400 MHz, CD_2_Cl_2_, 25 °C): *δ* = 7.66 (bs, 2H, *H*_2_NEt_2_), 5.86 (s, 2H, Pyr^3,4^), 3.38–3.34 (m, 2H, CH_2_P), 3.30 (q, *J* = 7.4 Hz, 4H, NC*H*_*2*_CH_3_), 2.59–2.44 (m, 4H, C*H*CH_3_), 1.46 (t, *J* = 7.3 Hz, 6H, NCH_2_C*H*_*3*_), 1.39–1.22 (m, 24H, CHC*H*_*3*_) ppm; ^13^C{^1^H} NMR (101 MHz, CD_2_Cl_2_, 25 °C): *δ* = 138.4 (C_q_, Pyr^2,5^), 108.2 (Pyr^3,3^), 42.5 (N*C*H_2_CH_3_), 25.8 (t, *J* = 7.6 Hz, *C*HCH_3_), 24.7 (*C*H_2_P), 19.7 (CH*C*H_3_), 19.3 (CH*C*H_3_), 11.5 (NCH_2_*C*H_3_) ppm; ^31^P{^1^H} NMR (162 MHz, CD_2_Cl_2_, 25 °C): *δ* = 49.9 ppm.

## X-ray structure determination

X-ray diffraction data of **5**,** 7**, **11**, and **12**⋅CH_2_Cl_2_ (CCDC 2301968, 2301969, 2301970 and 2301972) were collected at *T* = 100 K in a dry stream of nitrogen on a Bruker Kappa APEX II diffractometer system using graphite-monochromatized Mo-*K*α radiation (*λ* = 0.71073 Å) and fine sliced *φ*- and *ω*-scans. Data were reduced to intensity values with SAINT and a correction for absorption effects was applied with the multi-scan approach followed by a spherical absorption correction using SADABS or TWINABS [32]. The structures were solved by the dual-space approach implemented in SHELXT [33] and refined against *F*^2^ with SHELXL [34]. Non-hydrogen atoms were refined with anisotropic displacement parameters. H atoms attached to C were placed in calculated positions and thereafter refined as riding on the parent atoms. The positions of the ammonium hydrogen atoms in **12** were refined freely. Crystals of **5** were systematically twinned by reflection at (1–10) owing to local pseudo-symmetry. Molecular graphics were generated with the program MERCURY [35].

## Computational details

The computational results presented have been achieved in part using the Vienna Scientific Cluster (VSC). Calculations were performed using the Gaussian 09 software package [36] with the PBE0 functionals without symmetry constraints, the Stuttgart/Dresden ECP (SDD) basis set to describe the electrons of titanium, zirconium, and hafnium and a standard 6-31G** basis for all other atoms as already described previously [24].

### Supplementary Information

Below is the link to the electronic supplementary material.

Supplementary file1 (CIF 7792 KB)

## Data Availability

All relevant data are included in the manuscript.

## References

[1] Gossage RA, van de Kuil LA, van Koten G (1998). Acc Chem Res.

[2] Albrecht M, van Koten G (2001). Angew Chem Int Ed.

[3] van der Boom ME, Milstein D (2003). Chem Rev.

[4] Singleton JT (2003). Tetrahedron.

[5] Liang LC (2006). Coord Chem Rev.

[6] Morales-Morales D, Jensen CM (2007). The chemistry of pincer compounds.

[7] Nishiyama H (2007). Chem Soc Rev.

[8] Benito-Garagorri D, Kirchner K (2008). Acc Chem Res.

[9] Choi J, MacArthur AHR, Brookhart M, Goldman AS (2011). Chem Rev.

[10] Selander N, Szabo KJ (2011). Chem Rev.

[11] Bhattacharya P, Guan H (2011). Comments Inorg Chem.

[12] Schneider S, Meiners J, Askevold B (2012). Eur J Inorg Chem.

[13] van Koten G, Milstein D (2013). Organometallic pincer chemistry.

[14] Szabo KJ, Wendt OF (2014). Pincer and pincer-type complexes applications in organic synthesis and catalysis.

[15] Asay M, Morales-Morales D (2015). Dalton Trans.

[16] Murugesan S, Kirchner K (2016). Dalton Trans.

[17] Moulton CJ, Shaw BL (1976). J Chem Soc Dalton Trans. 1020

[18] Merz LS, Ballmann J, Gade LH (2020). Eur J Inorg Chem.

[19] Gruger N, Wadepohl H, Gade LH (2022). Dalton Trans.

[20] Kumar S, Mani G, Mondal S, Chattaraj PK (2012). Inorg Chem.

[21] Venkanna GT, Ramos TVM, Arman HD, Tonzetich ZJ (2012). Inorg Chem.

[22] Sekiguchi Y, Meng F, Tanaka H, Eizawa A, Arashiba K, Nakajima K, Yoshizawa K, Nishibayashi Y (2018). Dalton Trans.

[23] Idelson C, Webster L, Krämer T, Chadwick FM (2020). Dalton Trans.

[24] Tomsu G, Stöger B, Kirchner K (2023). Organometallics.

[25] Weng W, Yang L, Foxman BM, Ozerov OV (2004). Organometallics.

[26] Brammell CM, Pelton EJ, Chen C-H, Yakovenko AA, Weng W, Foxman BM, Ozerov OV (2011). J Organomet Chem.

[27] Liang LC, Chien PS, Hsiao YC, Li CW, Chang CH (2011). J Organomet Chem.

[28] Fryzuk MD, Carter A, Rettig SJ (1992). Organometallics.

[29] Sietzen M, Batke S, Antoni PW, Wadepohl H, Ballmann J (2017). Dalton Trans.

[30] Perrin DD, Armarego WLF (1988). Purification of laboratory chemicals.

[31] Kessler JA, Iluc VM (2014). Inorg Chem.

[32] Bruker computer programs (2020). APEX3, SAINT SADABS.

[33] Sheldrick GM (2015). Acta Crystallogr A.

[34] Sheldrick GM (2015). Acta Crystallogr C.

[35] Macrae CF, Edgington PR, McCabe P, Pidcock E, Shields GP, Taylor R, Towler M, van de Streek J (2006). J Appl Cryst.

[36] Frisch MJ, Trucks GW, Schlegel HB, Scuseria GE, Robb MA, Cheeseman, Scalmani G, Barone V, Mennucci B, Petersson GA, Nakatsuji H, Caricato M, Li X, Hratchian HP, Izmaylov AF, Bloino J, Zheng G, Sonnenberg JL, Hada M, Ehara M, Toyota K, Fukuda R, Hasegawa J, Ishida M, Nakajima T, Honda Y, Kitao O, Nakai H, Vreven T, Montgomery JA, Peralta JE, Ogliaro F, Bearpark M, Heyd JJ, Brothers E, Kudin KN, Staroverov VN, Kobayashi R, Normand J, Raghavachari K, Rendell A, Burant JC, Iyengar SS, Tomasi J, Cossi M, Rega N, Millam JM, Klene M, Knox JE, Cross JB, Bakken V, Adamo C, Jaramillo J, Gomperts R, Stratmann RE, Yazyev O, Austin AJ, Cammi R, Pomelli C, Ochterski JW, Martin RL, Morokuma K, Zakrzewski VG, Voth GA, Salvador P, Dannenberg JJ, Dapprich S, Daniels AD, Farkas Ö, Foresman JB, Ortiz JV, Cioslowski J, Fox DJ (2009). Gaussian 09, revision A.02.

